# A streamlined collecting and preparation protocol for DNA barcoding of Lepidoptera as part of large-scale rapid biodiversity assessment projects, exemplified by the Indonesian Biodiversity Discovery and Information System (IndoBioSys)

**DOI:** 10.3897/BDJ.5.e20006

**Published:** 2017-10-31

**Authors:** Olga Schmidt, Axel Hausmann, Bruno Cancian de Araujo, Hari Sutrisno, Djunijanti Peggie, Stefan Schmidt

**Affiliations:** 1 SNSB - Zoologische Staatssammlung München, Munich, Germany; 2 Division of Zoology, Research Center for Biology, Indonesian Institute of Sciences, Cibinong, Indonesia

**Keywords:** Collecting methods, DNA barcoding, light trap, monitoring, moths, rapid biodiversity assessment, sampling protocol

## Abstract

Here we present a general collecting and preparation protocol for DNA barcoding of Lepidoptera as part of large-scale rapid biodiversity assessment projects, and a comparison with alternative preserving and vouchering methods. About 98% of the sequenced specimens processed using the present collecting and preparation protocol yielded sequences with more than 500 base pairs. The study is based on the first outcomes of the Indonesian Biodiversity Discovery and Information System (IndoBioSys). IndoBioSys is a German-Indonesian research project that is conducted by the Museum für Naturkunde in Berlin and the Zoologische Staatssammlung München, in close cooperation with the Research Center for Biology – Indonesian Institute of Sciences (RCB-LIPI, Bogor).

## Introduction

Large-scale biodiversity inventory projects are becoming increasingly popular (see Janzen et al. 2009, deWaard et al. 2009, Basset et al. 2012, Tänzler et al. 2012, Hausmann et al. 2013, Telfer et al. 2015, Aagaard et al. 2016, Geiger et al. 2016, Miller et al. 2016, Wilson et al. 2016), particularly after the method of DNA barcoding has been established as a fast and efficient species discovery and identification tool (see Hebert et al. 2003, Packer et al. 2009, Ratnasingham and Hebert 2013).

The present paper introduces the ongoing large-scale biodiversity discovery project IndoBioSys (Indonesian Biodiversity Discovery and Information System). The project is focusing on establishing a vertebrate and invertebrate diversity discovery pipeline and a biodiversity information system in Indonesia. Samples are processed through an integrated sorting pipeline that has been set up and optimized at the Zoologische Staatssammlung in Munich, Germany, and tested at the Museum Zoologicum Bogoriense in Cibinong, Indonesia. For DNA barcoding, samples were submitted to the Canadian Centre for DNA Barcoding in Guelph, Canada.

A targeted, biodiversity knowledge-based screening approach for the identification of novel active biological compounds is a central aspect of the project. For this purpose, the initiative aims at providing a comprehensive biodiversity inventory, including descriptions of new species with sequence data that are publicly available in the Barcode of Life Data Systems (BOLD) and also integrated in the Indonesian Biodiversity Information System (IBIS), aimed at providing access to existing and new information on Indonesia’s biological resources. The present paper focuses on the insect order Lepidoptera that is among the largest and economically most important groups of insects.

Although general collection protocols compiled for molecular studies are available (e.g. http://lepbarcoding.org/protocols.php, Hajibabaei et al. 2005, Ivanova and Kuzmina 2013), they are rather standard and focused mostly on the pipelines for the DNA sequencing data analyses using already available material (i.e. pinned or papered museum specimens and field samples). Considering that DNA sequencing techniques are advanced and well-established but strongly depend on the quality of specimens, our collection protocol helps overcoming challenges in obtaining high-quality samples suitable for both morphological and DNA analyses and presents a workflow that secures availability of tissues, abstracts and data for future studies.

**Figure 1.**
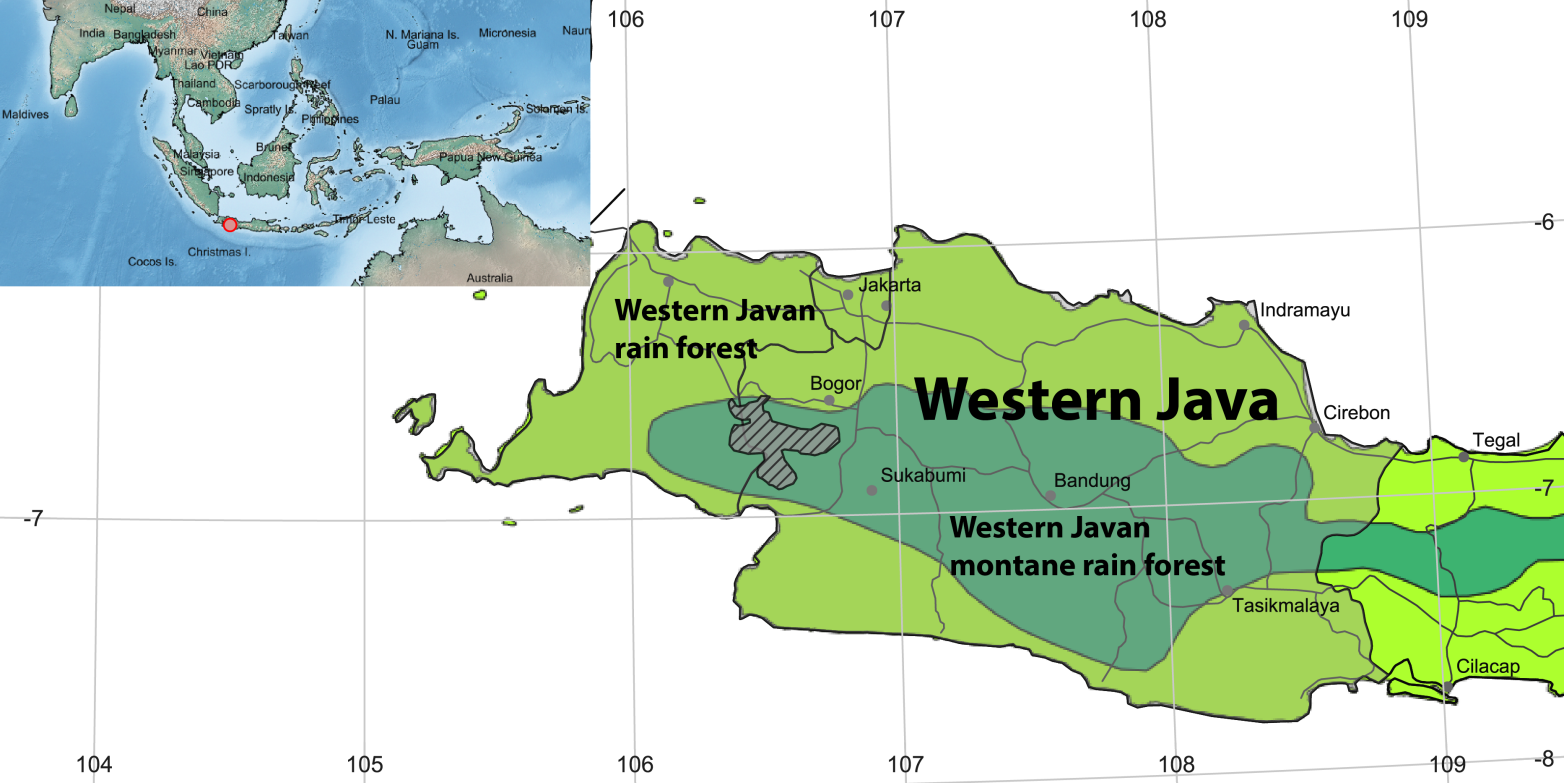
Map of Western Java showing the Halimun-Salak national Park (hatched). The study area is located in the Western Javan montane rain forest ecozone (dark green). Red dot in inset map shows the location of the study area in the Sundaland region. Map created with SimpleMappr (http://www.simplemappr.net).

**Figure 2.**
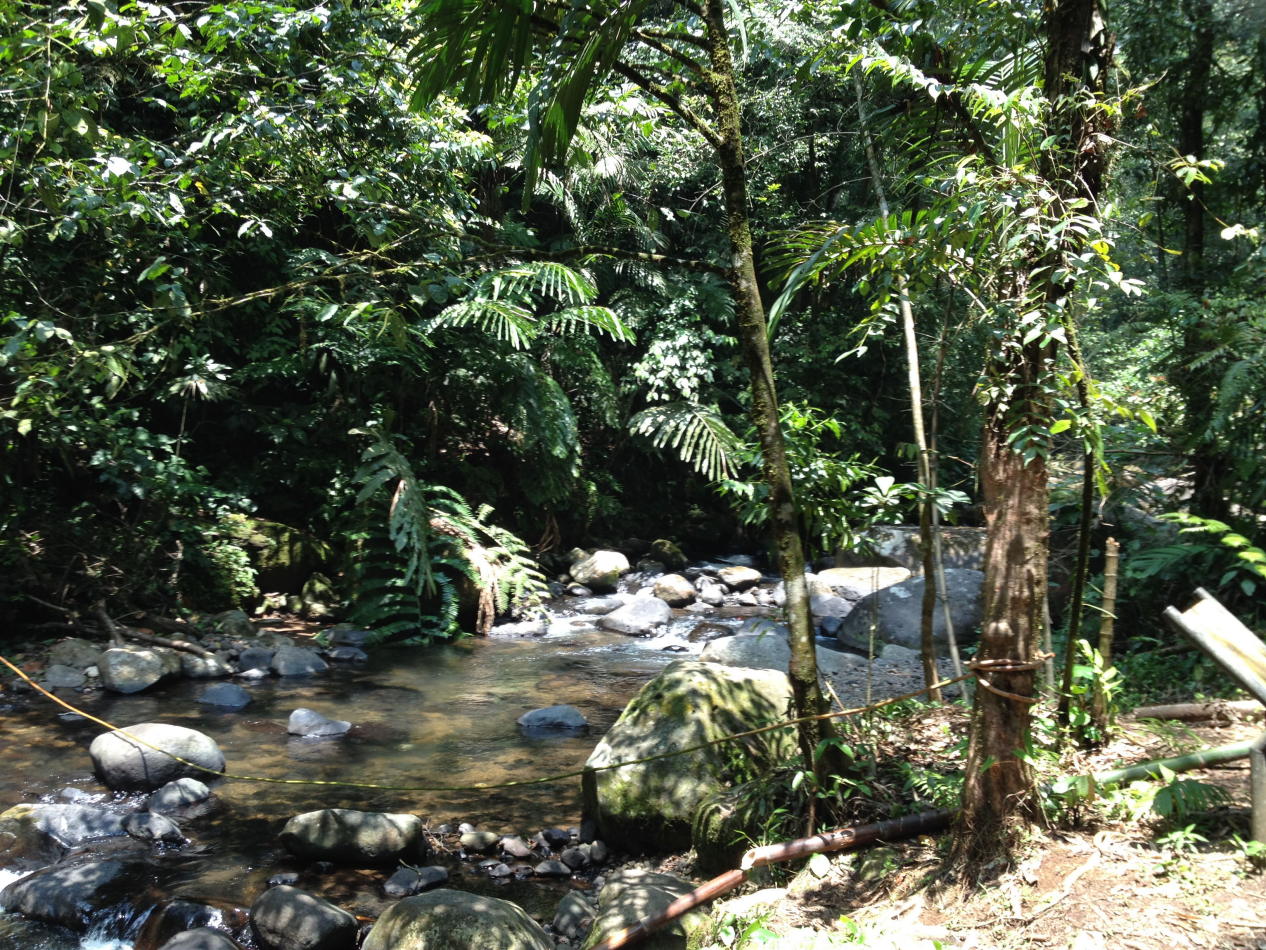
Halimun National Park (Indonesia, West Java), one of the collecting sites.

The highly diverse biota of Indonesia comprise both Oriental and Australian elements, with a high proportion of endemic species (see Holloway 1985, Holloway 1994, Holloway 1996, Holloway 1997, Schmidt 2005, Schmidt 2013, Schmidt 2015). The IndoBioSys study area, where extensive sampling has been carried out, is located in the Mount Halimun-Salak National Park, a conservation area in the Indonesian province of West Java (Fig. 1). Covering 113,357 hectares, the National Park harbours the largest sub-mountain forest in West Java (Fig. 2). The vegetation comprises primary and secondary forest and cultivated areas, with primary forest covering almost 70% of the area (Hartono et al. 2007).

Moths of Indonesia are poorly studied (see Schmidt 2015) and no checklists of moths occuring in Indonesia have been published. Some ecological studies have been conducted on Indonesian Lepidoptera, including several recent surveys of Macrolepidoptera in secondary forests, national parks, protected forests and mountane populated areas of Maluku Islands, West Papua, Central Kalimantan, and Java. As a result, lists of Macrolepidoptera have been compiled, comprising 160 species from 14 families collected on the island of Ternate, 178 species from 19 families collected at the base of the Foja Mountain Nature Reserve (Sutrisno 2012), up to 214 species from 21 families on different plots of Gunung Patuha protected forest (Sutrisno 2009), up to 278 species from 19 families on different plots of Central Kalimantan (Sutrisno 2005), up to 297 species from 19 families in the Nusa Barong Nature Reserve (Sutrisno 2007), and up to 846 species on different plots of Halimun-Salak National Park (Sutrisno 2008). No definite conclusions have been drawn about the total number of species in certain localities as further studies were needed (see Sutrisno 2005, Sutrisno 2007, Sutrisno 2012). Many undescribed species were expected to occur in the study areas (see Sutrisno 2005) but no new species were formally described, and the identification of specimens was mainly based on external morphological characters and study of the types was beyond the frame of these primarily environmental studies. It is highly probable that sibling species may have been overlooked (Schmidt, unpubl. data). For comparison, biodiversity studies conducted on Borneo revealed more than 1,000 species of Geometridae in this area (Holloway 1994, Holloway 1996, Holloway 1997). Considering the country size and diversity of habitats, Indonesia is expected to be one of the main biodiversity hotspots, and further biodiversity assessment studies based on DNA barcoding would rapidly increase the knowlege of the largely unknown Indonesian moth fauna.

## Material and methods

Sampling of Lepidoptera has been conducted at three plots in the study area at different elevations during one dry and one wet season. Material has been collected during the day using a sweep net and at night using standard light sources having a strong emission in the ultraviolet range of the spectrum. At each plot, two light traps at a distance of about 30-40 meters from each other were operated simultaneously: (1) a UV light trap with two light sources, including black light lamps and fluorescent light tubes (8 W each) running from rechargeable 12 V batteries, (2) a mercury vapour light trap (125 W) running from a generator. The light sources were placed in front of a white sheet and protected from the rain by an umbrella. Active sampling (no killing traps) was preferred. Moths were one of the major target groups for the project. Collection- and general preparation methods, as well as digital imaging of specimens, have been described in numerous studies (e.g. Common and Waterhouse 1972, Klots 1973, Common 1990, Landry and Landry 1994, Prendini et al. 2002, Häuser et al. 2005, Paulson 2005, Gibb and Oseto 2006, Krogmann and Holstein 2010, Infusino et al. 2017) and in various contributions on the web (e.g. Wheeler et al. 2001, Warren 2015). Obtained specimens were treated according to the barcoding protocol developed in the Biodiversity Institute of Ontario (Guelph, Canada) (Ivanova et al. 2006, Wilson 2012) and are included in BOLD (Ratnasingham and Hebert 2007).

### Collecting equipment

1. Light sources/bulbs, electricity/power sources (generators/accumulators).
2. Torches
3. White sheet, ca. 200x300 cm, alternatively light tower/light tent constructions
4. Rope and pegs to hang the white sheet (see Schmidt (2016): fig. 1)
5. Collecting net
6. A large number of killing bottles of different sizes (e.g. with potassium cyanide, KCN) filled with a few narrow stripes of crumpled filter paper and/or glass vials with cork stoppers
7. Forceps for handling stripes of filter paper
8. Syringe and ammonium chloride for killing larger Lepidoptera (and keeping them relaxed)
9. GPS receiver
10. A tool kit for setting up light traps

### Equipment for preservation

1. Entomological pins
2. Featherweight and fine-point entomological forceps
3. A pair of scissors
4. Labels for sample data
5. Pencils and marker pens
6. Envelopes with layers of cotton in a plastic container
7. Well closing boxes with plastazote foam bottom for pinned Lepidoptera
8. Orange silica gel
9. Relaxing boxes
10. Setting boards, strips of grease-proof paper and setting pins
11. Gelatine capsules (for preserving a Lepidoptera leg prior to relaxing a specimen)

### Equipment for DNA barcoding

1. Ethanol (96%), pipettes for transferring one drop each into the tubes of the lysis plates
2. Lysis plates fitted with cap-stripes for processing of DNA barcoding samples
3. Featherweight and fine-point entomological forceps for leg-picking and mounting of tissues in lysis plate wells
4. Specimen labels with DNA Barcoding sample IDs
5. Computer for capture of specimen data
6. Camera for photography of voucher specimens

## Collection protocol

### Fieldwork. Specimen sampling

- During the day: Collect specimens in a killing bottle using net-sweeping. Attach labels to the samples containing information on the locality (country, province, region), the GPS data, the altitude, the date and name of collectors and collecting methods. Make field notes. **Note**: (1) Numerous groups of moths are active in the afternoon/evening or are readily flushed from the vegetation and may be sampled using net-sweeping. (2) If the aim of the project is to achieve close-to-complete biodiversity inventories, additional methods are needed, e.g. bait, malaise traps, collecting of larval stages (e.g. many small Microlepidoptera like leaf-rollers, leaf-miners etc.).
- At night: Collect moths in a large number of smaller/medium killing jars. In good collecting nights with many moths, after 3-5 minutes transfer them to larger killing jars to get the smaller jars free. After 20-30 minutes (when dead) transfer them from large sample jars to cotton sheets carefully using featherweight forceps, moths should not overlap. Keep these cotton layers in a well closing box (against ants and other pests) in a cool, dry place until next morning. Bring silica gel into the box. Attach labels to the samples and make field notes. Larger moths may be killed with ammonium chloride injected with a syringe. Alternatively, sample small-sized moths in small glass vials/tubes with cork stoppers, keep them alive overnight in a cool place and mount next day. **Note**: (1) In case of a teamwork at a stationary light trap, it is possible to pin at least part of killed specimens immediately. (2) Some groups of moths (e.g. Geometridae) come to light at night and stay not only on a white sheet but also sit on the leaves of trees and bushes near the light trap.
- Next morning: Change silica gel in the boxes with cotton layers (if necessary). If the collector is experienced, check all the collected specimens, trying to group the sample by morphospecies. Mount (pin) 3-4 representatives of each morphospecies group (in certain projects with large sample sizes it may be recommendable to focus this step on target groups). Each morphospecies group should be documented, including photographs. Carefully label all cotton layers and all pinned specimens (ad interim this can be made collectively for batches). Create field numbers for further use. **Note**:(1) In case of a teamwork it is possible to spread the wings of at least part of freshly collected specimens. Keep the spreading boards in containers and change orange silica gel in time. Specimens can also be dried more quickly in an oven set to a low temperature (ca. 50˚C). (2) A labelling protocol based on Quick Response (QR) codes was implemented to accelerate and facilitate labelling of samples in the field.

### Post-fieldwork

- Make a general photograph of each cotton layer with labels, which will help sorting and selecting specimens for further study.
- Prepare locality- and (if possible) identification labels and label all the pinned specimens.
- Change the orange silica gel in plastic containers regularly until the collected specimens get dry.
- Convert handwritten field notes into digital form. Organize and secure the digital data.
- If spreading of the wings is required before the pre-lab preparation of the specimens, remember that relaxing of the specimens using a relaxing box will destroy the DNA. The following procedure is recommended. Remove two legs prior to relaxation of a specimen, place the legs in a gelatine capsule, pin an identical provisional number under the capsule and the specimen. Spread the wings of the specimens using spreading boards. Remove dried specimens from the spreading board and pin the gelatine capsule containing legs under the specimen, along with the proper labels. **Note**: Two samples (legs) were removed from each voucher in case the first sample fails in which case the barcode analysis can be repeated with the second sample.

### Pre-lab preparation protocol

- Select 95 specimens with locality labels for a lysis plate and pin a number (sample ID) under each specimen. **Note**: Make sure that each specimen is assigned a unique sample identification number that will be recorded in the CCDB data record spread sheet.
- Pin 95 selected labelled specimens in a separate insect box for further action.
- Make a first photograph of each of the 95 specimens (following the photo guidelines of BOLD) and save files according to the instructions for submission.
- Enter required data to the BOLD spreadsheets. **Note**: 96-well plates are delivered with detailed instructions for data submission (see http://ibol.org/wp-content/uploads/2014/07/Instructions_PCR.pdf). Sign the BMAA (Biological Material Analysis Agreement) prior to shipping the plates to the Canadian Centre for DNA barcoding.
- One by one break a middle right leg of each of the 95 specimens, place inside 95 wells of the barcoding plate. A drop of 96% Ethanol should be added in each well to avoid electrostatic problems during tissue sampling and during re-opening of the plates prior to DNA-extraction. Fix the stripes. **Note**: Leave the 96^th^ well empty for negative control.
- Pack 96-well plates and send them to the Biodiversity Institute of Ontario for further study (Address: Sample Submission, Dr P.D.N. Hebert, Centre for Biodiversity Genomics, Biodiversity Institute of Ontario, University of Guelph, 50 Stone Road East, Guelph, Ontario, Canada N1G 2W1, Phone: +1-519-824-4120 ext. 58259). Submit four sets of data: (1) Completed BOLD Specimen Data Template, including the voucher info, taxonomy, specimen details and collection data (submit to BOLD, http://www.barcodinglife.org); (2) ImageData spread sheet (submit to BOLD, http://www.barcodinglife.org); (3) Images of 95 specimens (submit to BOLD, http://www.barcodinglife.org), and (4) CCDB Plate Record sheet (submit to LIMS@ccdb.ca). **Note**: If you want to recover tissues or whole specimens (e.g. when whole bodies need to be extracted because of the minute size of the voucher) after the DNA-extraction, make the following note on a barcoding plate, ‘Voucher Recovery Plate’).

### Post-lab activity. Storing of the vouchers

All the vouchers should be deposited in a public insect collection and stored in insect drawers in entomological cabinets under proper conditions to protect them from climatic conditions and insect pest attacks as the voucher specimens are linked to the DNA barcode reference library and establish a base for testing and verification of the results.

### Frequently asked questions

The present collecting and preparation protocol is a manual used by the students, technical staff and researchers involved in the study. Here, we provide responses to some of the more common issues raised.

- We did not employ automatic traps because they do not yield well-preserved, high-quality material suitable for morphological studies.
- We did not specifically study the impact of long-term preservation of Lepidoptera in ethyl acetate. The specimens were killed with ethyl acetate vapours, removed from the killing jars after about 20-30 minutes and successfully used for the DNA analysis.
- We did not use glassine envelopes for storage of single specimens. Instead, we used medium-sized envelopes with layers of cotton in a plastic container to save time while preserving freshly collected material.
- We make sure that each specimen is assigned a unique sample identification number before entering required data to the BOLD spreadsheets to avoid possible confusion.
- We make a photograph of a specimen before entering required data to the BOLD spreadsheets and leg picking to document the specimen as soon as possible, in (an improbable) case the specimen gets damaged.
- We take special care when storing the voucher specimens as they are linked to the DNA barcode reference library and establish a base for testing and verification of the results.

## Results and discussion

**Figure 3.**
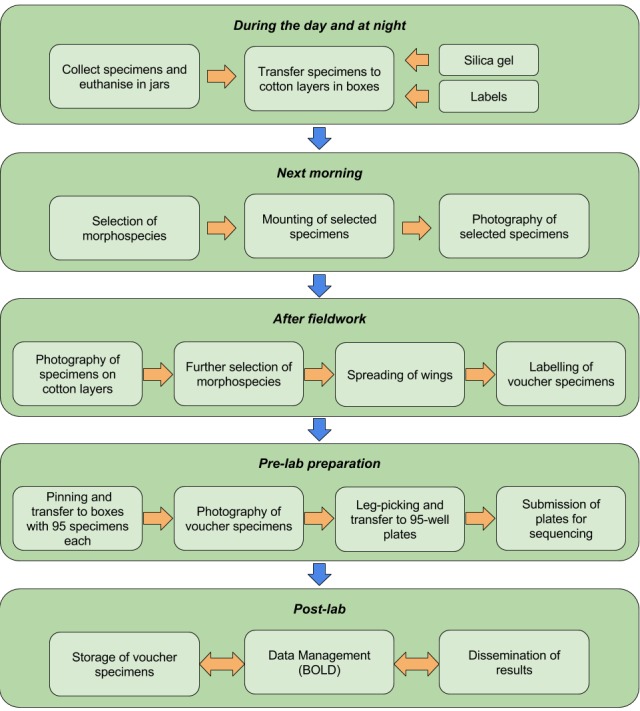
The workflow from collecting to storage of specimens of Lepidoptera in our IndoBioSys project.

**The workflow.** The collection protocol presented in this article has been successfully employed for field- and pre-lab activities that were part of the IndoBioSys project in the years 2015 and 2016. The workflow is presented in Fig. 3. During the first stage of the survey of the Indonesia’s Lepidoptera diversity we focused on a few target groups, including the Geometridae. More than thirty 96-well lysis plates that are routinely used for DNA barcoding by the Canadian Centre for DNA Barcoding (CCDB) were processed using a high-throughput protocol, and several plates were additionally processed at the Zoologische Staatssammlung (ZSM, Munich).

**Success rates.** About 98% of the sequenced specimens of the Geometridae processed using the present collecting and preparation protocol yielded sequences with more than 500 base pairs, meeting the length requirement for DNA barcode status (see Ratnasingham and Hebert 2007). When other protocols were implemented (e.g. for Malaise trap samples or samples stored without using sufficient amounts of silica gel) less than 88% of the specimens yielded sequences with more than 500 base pairs. Performing a statistical analysis to compare different protocols is beyond the scope of the present study.

**Treatment of specimen with ethyl acetate.** Sequencing for inventory projects like IndoBioSys requires killing and preserving the specimens in a DNA-friendly way. The Lepidoptera were killed with ethyl acetate vapours and in most cases removed from the killing jars after about 20-30 minutes (but not longer than 40 minutes) to make sure that the DNA is not damaged. Our results confirm the findings by Willows-Munro and Schoeman (2014) suggesting that ethyl acetate can be successfully used to collect specimens for DNA analysis. However, the impact of long-term preservation of Lepidoptera in ethyl acetate has not been studied.

**Remarks to a recently publised alternative preserving and vouchering method.** In a recent publication by Cho et al. (2016) a procedure for preserving and storing Lepidoptera tissues has been presented. This procedure aims at creating accessible and easily visualized “wing vouchers” of individual Lepidoptera specimens while preserving the remainder of the insect in a cryogenic freezer for molecular research, with the wings preserved in protective plastic holders so that both dorsal and ventral patterns and colours can be easily viewed. However, this method involves removing a pair of wings (in some cases all wings) from a specimen. It is not just the “aesthetically pleasing display” that matters. Scissors are used to cut the wings at the base, whereby the wing base gets damaged which hampers the study of the morphological characters (e.g. venation in both sexes and androconial scales in males). Besides, this procedure is obviously not recommended for treatment of type specimens, considering the amount of undescribed rare taxa processed while conducting research related to a large-scale biodiversity discovery projects in a diverse and poorly studied region. Another drawback of the “wing vouchering” approach is its inapplicability to small moths (Cho et al. 2016). Our proposed method does not suffer from these drawbacks because we remove legs from the freshly collected specimens and preserve them for molecular analysis. An important part of the procedure is that the tissues get dry as fast as possible, either in a well closing box with silica gel beads or in a drying oven. Our method is applicable to all Lepidoptera.

Additionally, reliable storage methods were described and discussed by Knölke et al. (2005) and Lopez-Vaamonde et al. (2012).

**Towards future outcomes.** The ultimate objective of our study is to present a methodological pipeline assisting in successful sampling, preparation, preservation, morphological and molecular analyses and secure storage of high-quality material for a biodiversity assessment which combines the expertise gained through the DNA barcoding and the taxonomist’s knowledge.
